# Phenanthrene degradation and biosurfactant production by *Stenotrophomonas maltophilia* under static conditions: potential for bioremediation

**DOI:** 10.3389/fbioe.2026.1983589

**Published:** 2026-10-21

**Authors:** Sreenidhi Yuvaraja, Tishrota Chakraborty, Jayashree Gomathysankar, Sakthishabarish Kumar, Kannabiran Krishnan

**Affiliations:** School of Biosciences and Technology, Vellore Institute of Technology, Vellore, India

**Keywords:** biodegradation, biosurfactants, phenanthrene, phytotoxicity, polycyclic aromatic hydrocarbon

## Abstract

Phenanthrene, a polycyclic aromatic hydrocarbon, poses significant ecological risks due to its hydrophobicity and environmental persistence. This study explores phenanthrene degradation and biosurfactant production under static conditions by the isolate VITSVK4 from uncontaminated terrestrial soil. The isolate, identified as *Stenotrophomonas maltophilia* based on 16S rRNA gene sequencing, achieved 72% ± 1.73% degradation of 100 mg/L phenanthrene and exhibited strong biosurfactant production with an emulsification index of 79.2% ± 2.51%. The strain tolerated higher phenanthrene concentrations and maintained 59.3% ± 2.08% degradation at 500 mg/L over 8 days under static conditions and GC-MS analysis confirmed degradation of phenanthrene. Seed germination studies indicated reduced phytotoxicity on degradation; phytotoxicity of phenanthrene on *Vigna radiata* for treated and untreated phenanthrene was 24.5% and 94.7%, respectively. Treated phenanthrene showed 13.1% phytotoxicity on *Triticum aestivum*. In addition, *S. maltophilia* degraded crude oil, used engine oil, and monoaromatic petroleum hydrocarbons and demonstrated 60% ± 2.51% oil removal in oil recovery assay. FTIR analysis indicated a glycolipid type biosurfactant that remained stable across varying pH, temperature, and salinity. These results highlight phenanthrene degradation and biosurfactant production under static conditions as a promising strategy for petroleum hydrocarbon remediation.

## Graphical Abstract

**Figure:**
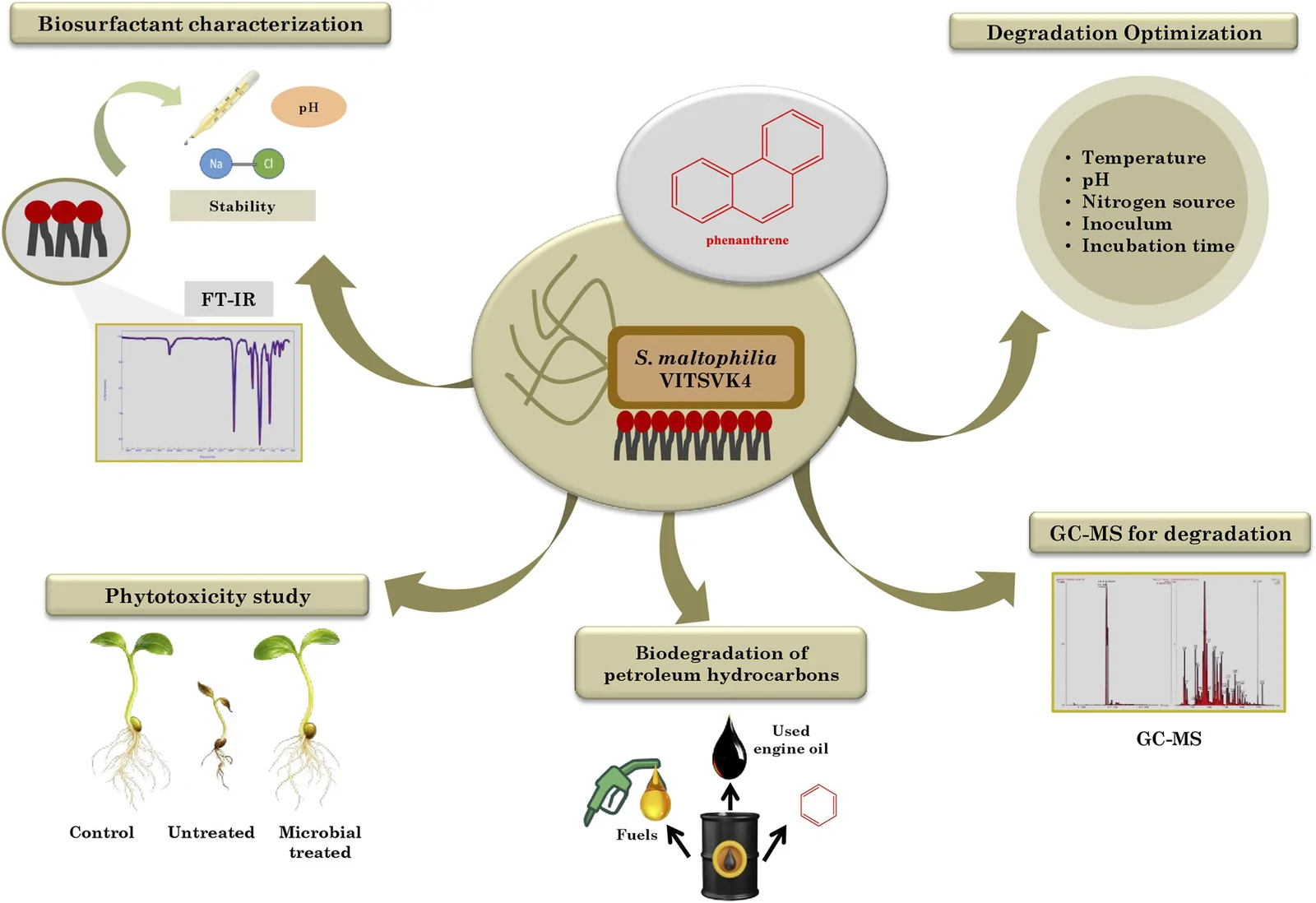
Phenanthrene degradation and biosurfactant production by S. maltophilia VITSVK4.

## 1 Introduction

The rising demand for petroleum derived products has increased global production and transportation, and this growth of the petrochemical industry has increased the likelihood of petroleum hydrocarbon contamination (Duarte et al., 2024). Pollutants enter the environment through automobile exhaust, refinery emissions, accidental spills and leakages during refining, transportation, and storage of petroleum products (Imam et al., 2021). Contamination of petroleum hydrocarbons alters ecosystems, affecting environmental and human health (Mekonnen et al., 2024). Phenanthrene, a polycyclic aromatic hydrocarbon (PAH), is classified as a persistent organic pollutant. It has a tricyclic structure and is used as a model PAH compound due to its unique structure, moderate toxicity, and widespread distribution (Li et al., 2025). Phenanthrene is toxic, mutagenic, and carcinogenic, which can potentially cause adverse effects to plants, animals, and humans. Effective microbial removal of phenanthrene is important, as it exhibits toxicity, low solubility, and most microbes do not possess the potential to degrade phenanthrene (Sumathi and Manian, 2024).

Bioremediation utilizes the ability of microbes to transform pollutants into less or non-toxic forms. It is described as a sustainable and cost effective approach and differs from physicochemical methods that can be expensive and may generate secondary pollutants (Raheema et al., 2026). Biodegradation is an effective strategy for the remediation of petroleum hydrocarbon contamination. Numerous bacterial genera, including *Achromobacter, Acinetobacter, Alcanivorax, Bacillus, Marinobacter, Micrococcus, Pseudomonas,* and *Rhodococcus,* have been reported for their capacity to degrade petroleum hydrocarbons (Shokranian et al., 2025). Biosurfactants are surface active compounds produced by microbes and are reported as effective and sustainable agents to enhance petroleum hydrocarbon bioremediation (Zahed et al., 2022). These amphiphilic molecules improve the mobility and solubility of hydrophobic compounds by reducing interfacial and surface tension (Markam et al., 2024). Biosurfactants exhibit the potential to sustain activity in adverse environments, such as high salinity, extreme temperatures, or varying pH, commonly observed at contaminated sites. Such versatility makes biosurfactants a suitable alternative to chemical surfactants, which can be toxic and recalcitrant in the environment (Mandalenaki et al., 2021).

*Stenotrophomonas maltophilia*, a metabolically diverse Gram-negative bacterium widely distributed in various environmental habitats, has garnered significant attention for its potential applications in bioremediation and its potential to degrade xenobiotics, including petroleum hydrocarbons, has been reported (Mukherjee and Roy, 2016). The capacity of *Stenotrophomonas maltophilia* to degrade PAHs, including phenanthrene, has been demonstrated through various studies. *Stenotrophomonas maltophilia* RC was reported to degrade phenanthrene and anthracene (Chowdhury et al., 2017)*.* Acidophilic *S. maltophilia* strain AJH1 was reported to degrade phenanthrene, anthracene, naphthalene, and high molecular weight PAHs (Arulazhagan et al., 2017). *Stenotrophomonas maltophilia* CPHE1 degrades and mineralizes phenanthrene (Lara-Moreno et al., 2021). *Stenotrophomonas maltophilia* strain VUN 10,003 degrades coronene, benzo [a]anthracene, fluoranthene, benzo [a]pyrene, and pyrene (Juhasz et al., 2000), and *S. maltophilia* JPHC3Z2B degrades phenanthrene (Nzila et al., 2018).

Studies on bioremediation of phenanthrene are well established, but research on evaluating the biodegradation of phenanthrene and its biosurfactant potential under static (non-agitated) conditions is limited. Biodegradation under such conditions is significant, as many bioremediation approaches typically study degradation under well aerated, agitated conditions, which do not represent real contaminated sites. Under these conditions, bacteria with the ability to degrade under static conditions can effectively remediate petroleum hydrocarbons. This study focuses on the potential of *S. maltophilia* VITSVK4, isolated from uncontaminated terrestrial soil, to degrade phenanthrene under static conditions, and characterizes and evaluates the stability of its biosurfactant for bioremediation applications.

## 2 Methodology

### 2.1 Sample collection, phenanthrene stock and inoculum preparation

Uncontaminated terrestrial soil from Vallimalai hills (13.07°N, 79.26°E), Katpadi, Vellore, India was used for isolation. Soil was collected from a depth of 5–10 cm after removal of the exposed surface layer in a sterile container, transported within 24 h to the laboratory, and maintained at 4 °C until further processing. pH was measured at 6.78% ± 0.04%. Phenanthrene (98%) procured from HiMedia was used for degradation studies. 0.25 g of phenanthrene in 10 mL dichloromethane was used as a stock solution with 25 mg/mL concentration.

Isolates were grown in Bushnell Haas (BH) broth (HiMedia) with 0.1% glucose as the carbon source and incubated under static conditions for 48 h. Broth was centrifuged at 6000 rpm for 10 min at 4 °C, and the pellet was washed and resuspended in (BH) broth. The final OD was standardized to 1.0 at 600 nm and added at 1% (v/v) to the 50 mL working culture.

### 2.2 Enrichment and isolation

BH broth with 100 mg/L phenanthrene as the sole carbon source and 5 g of uncontaminated soil was used for enrichment culture, and the culture was maintained under static conditions for 7 days. Serial dilution was performed using 1 mL of enrichment broth, and the dilutions from 10^-4^–10^–6^ were spread on BH agar supplemented with 100 mg/L phenanthrene and incubated for 14 days. The bacterial colonies obtained after incubation were subcultured to obtain pure cultures (Ma et al., 2025).

### 2.3 Determination of phenanthrene biodegradation efficiency

50 mL of BH broth with 100 mg/L phenanthrene in 250 mL Erlenmeyer flasks was inoculated with the obtained isolates and incubated under static, non-agitated conditions for 7 days. 200 μL of phenanthrene stock was added to a culture working volume of 50 mL which correspond to 100 mg/L. Phenanthrene was added to the flasks and left in a laminar air flow chamber for 15 min to facilitate dichloromethane evaporation, followed by manual swirling for 2 min to resuspend phenanthrene. The nominal concentration was 100 mg/L, and the analytically measured concentration was 96.7 ± 1.9 mg/L. A sterilized, uninoculated abiotic control was maintained in 50 mL of BH broth with 100 mg/L phenanthrene and incubated under the same conditions to account for abiotic and non-biological loss. After incubation, the broth was centrifuged at 10000 rpm for 15 min. For both the abiotic control and biodegraded phenanthrene, liquid-liquid extraction was performed with hexane, and the organic phase was dried over anhydrous sodium sulphate. Erlenmeyer flasks were washed with hexane to extract phenanthrene adsorbed onto the flask surface. Phenanthrene was extracted with hexane with an extraction volume of 100 mL in three cycles and hexane had a recovery efficiency of 96.7% ± 1.9%. Biodegradation was calculated spectrophotometrically by measuring absorbance at 254 nm, the λ max of phenanthrene (Sumathi and Manian, 2024). Initial concentration from abiotic control and residual concentration from degraded broth was used for analysis. Samples exhibiting absorbance above the spectrophotometer’s linear range (>1) were diluted with hexane before measuring.

$$\text{Degradation }\left(\%\right)=\left[\left(\text{Initial Concentration}-\text{Residual concentration}\right)\;/\text{ Initial concentration}\right]\times 100$$

### 2.4 Biosurfactant screening

BH broth with 100 mg/L phenanthrene was inoculated with isolates and incubated for 7 days. The broth was subjected to centrifugation at 10000 rpm for 15 min at 4 °C. Triton X-100 was the positive control, distilled water was the negative control, and the following assays were performed. Screening assays were conducted in biological triplicates (n = 3).

#### 2.4.1 Oil spreading assay

Used engine oil (1 mL) was added to distilled water in a Petri dish, and supernatant (100 μL) was placed on the oil layer. The diameter of the clearance zone was measured (Ortiz-Lechuga et al., 2026).

#### 2.4.2 Drop collapse assay

Used engine oil (100 μL) was placed on a glass slide, and supernatant (25 μL) was added onto the drop of oil. The collapse of the drop was observed after 2 min (Shojaei and Mulligan, 2026).

#### 2.4.3 Emulsification index assay

The cell free supernatant (2 mL) was added to an equal volume of used engine oil, followed by vortexing. Emulsification index (E24) was calculated after 24 h (Ortiz-Lechuga et al., 2026).

$$\text{Emulsification index}=\left(\text{Height of emulsion layer}/\text{total height of solution}\right)\;x\;100$$

### 2.5 Biofilm formation screening (tube method)

The isolated strains were inoculated into nutrient broth and incubated at room temperature for 48 h, with an uninoculated broth serving as a negative control. Following incubation, the broth was discarded, and the tubes were washed with phosphate buffer (pH 7.4). The tubes were then stained with 0.1% crystal violet, incubated for 10 min, and washed with phosphate buffer. Biofilm formation was assessed visually, and visible staining along the tube walls was considered as positive (Harika et al., 2020).

### 2.6 Biofilm formation test (96 well method)

The nutrient broth culture of VITSVK4 was incubated for 24 h and diluted to 1:100 with M63 minimal medium containing arginine. 100 μL aliquot of the dilution was added to a 96-well microtiter plate and incubated for 24 h. The experiment was conducted in biological triplicates. Following incubation, the culture medium was discarded, and the wells were gently washed with phosphate buffer (pH 7.4). The adherent cells were then stained with 0.1% crystal violet, incubated for 15 min, and washed again with phosphate buffer. The plate was then inverted and allowed to air dry overnight. Crystal violet bound to the wells was solubilized in 30% acetic acid for 15 min, and absorbance at 550 nm was measured with a Microtiter plate reader, using 30% acetic acid as the blank. The results of three biological replicates were analyzed according to the criteria: Non-biofilm producer: OD ≤ ODc, weak biofilm producer: ODc < OD ≤ 2×ODc, moderate biofilm producer: 2×ODc < OD ≤ 4×ODc, and strong biofilm producer: 4×ODc < OD (Dhanasekaran et al., 2025).

$$\text{Cut off value }\left({\text{OD}}_{c}\right)=\text{Average of negative control OD}+3\times \text{standard deviation}$$

$$\text{OD}=\text{Average of test OD}-{\text{OD}}_{c}$$

### 2.7 Preliminary enzyme screening assays

#### 2.7.1 Manganese peroxidase assay

Nutrient agar containing 1% casein and 0.1% methyl red indicator was prepared. The isolates were streaked and incubated for 24 h. Yellow colour zone formation indicated the presence of manganese peroxidase enzyme (Kumari et al., 2022).

#### 2.7.2 Laccase assay

Nutrient agar (100 mL) was prepared with 12.5 μL of guaiacol and 0.12 g of CuSO_4_. The isolates were streaked and incubated for 24 h. Reddish-brown colour development upon oxidation of guaiacol indicates laccase production (Kumari et al., 2022).

#### 2.7.3 Lipase assay

The isolates were streaked over nutrient agar plates containing 1% tributyrin and 0.1% CaCl_2_ and incubated. The lipase enzyme produced by the bacteria results in clear halo zone formation around the colonies (Mahmood et al., 2017).

Based on the screening tests, the potential isolate was subjected to further studies.

### 2.8 Molecular characterization of VITSVK4 with 16S rRNA sequencing

The isolate VITSVK4 was identified with 16S rRNA gene sequencing to determine its molecular taxonomy and phylogenetic affiliation. Genomic DNA was extracted with high pure PCR template preparation kit (Roche). PCR amplification was performed with universal primers 27F and 1492R, followed by sequencing primers 518F and 800R. The resulting 16S rRNA gene sequence of VITSVK4 was compared with sequences in the GenBank database with BLAST analysis. The maximum composite likelihood model and Neighbor-joining distance method with 1000 bootstrap replications was used for phylogenetic tree construction. Phylogenetic tree construction and distance matrix analysis were performed using MEGA version 11 (Sakthishabarish et al., 2026).

### 2.9 Effect of nitrogen containing compounds on phenanthrene degradation

The effect of nitrogen containing compounds on phenanthrene degradation was determined by evaluating biodegradation with yeast extract, peptone, soybean meal, urea, ammonium sulphate, and ammonium nitrate. Ammonium nitrate, the nitrogen source in the standard composition of BH broth, was removed and replaced with each source at an equal weight of 0.1% (w/v) to evaluate biodegradation. The effect of yeast extract concentration was determined by estimating degradation with yeast extract at 0.1, 0.2, 0.3, 0.4, and 0.5% (w/v) for 7 days under static conditions. Sterilized, uninoculated abiotic control was maintained in 50 mL of BH broth with 100 mg/L phenanthrene, respective nitrogen containing compounds and incubated under the same conditions to account for abiotic and non-biological loss. (Zhu et al., 2016).

### 2.10 Estimation of growth and optimization

BH broth (50 mL) with 0.3% (w/v) yeast extract, 100 mg/L phenanthrene, and 1% inoculum (v/v) of VITSVK4 was incubated under static conditions, and biomass was weighed each day for 10 days. The broth was centrifuged at 10000 rpm for 15 min at 4 °C, and the cell pellet was washed with distilled water to remove media components and washed with hexane to remove residual phenanthrene before drying. The resulting pellet was dried, weighed, and the biomass was expressed in g/L (Soumeya et al., 2022).

The influence of different parameters on the degradation of phenanthrene by VITSVK4 was evaluated with one factor at a time (OFAT) method. The effects of temperature (27, 32, 37, and 42 °C), pH (5–9), inoculum volume (0.5%–3% v/v), and phenanthrene concentration (100–700 mg/L) were evaluated under static, non-agitated conditions for 8 days. Sterilized, uninoculated abiotic control was maintained in 50 mL of BH broth with phenanthrene, 0.3% yeast extract and incubated under the same conditions to account for abiotic and non-biological loss, and the degradation was estimated spectrophotometrically at 254 nm (Li et al., 2025).

### 2.11 Gas chromatography mass spectrometry

GC-MS analysis was performed to validate phenanthrene biodegradation. 50 mL of BH broth with 100 mg/L phenanthrene in 250 mL Erlenmeyer flasks was inoculated and incubated for 8 days under static, non-agitated conditions. Phenanthrene was extracted with hexane and subjected to analysis. Analysis was performed using PerkinElmer Clarus 680 and 600 with a capillary column (30 m, 0.25 mm ID, 0.25 μm df). The oven temperature was initially maintained at 60 °C for 2 min, ramped to 300 °C at 10 °C/min, and held for 6 min. Injector temperature was 260 °C, with an injection volume of 1 μL, and split injection (10:1). Helium at a constant flow rate of 1 mL/min was used as the carrier gas. The ion source and mass transfer line temperatures were maintained at 240 °C. Mass range was 40–600 Da, using electron ionization mode. Data acquisition and analysis were performed with Turbo Mass software version 5.4.2. Phenanthrene was identified by comparison of retention time and mass spectral pattern with NIST mass spectral library.

### 2.12 Seed germination test

The phytotoxicity of biodegraded phenanthrene, untreated phenanthrene, and distilled water (control) was evaluated with mungbean (*Vigna radiata*) and wheat (*Triticum aestivum*) seeds. Healthy and uniform seeds were selected and surface sterilized with 70% ethanol, 2% sodium hypochlorite, and distilled water (Agrawal and Shahi, 2017). Ten seeds were transferred onto 90 mm sterile Petri dishes lined with filter paper and the experiment was conducted in biological triplicates (n = 3). Each experimental dish received 3 mL of the respective treatment solution: (1) distilled water, (2) untreated phenanthrene solution (500 mg/L), and (3) biodegraded phenanthrene solution obtained after VITSVK4 degradation. The seeds were incubated at 25 °C ± 2 °C for 14 days. At the end of the study, number of seeds germinated was recorded, and shoot and root length were measured with a ruler (Wulandari et al., 2021). Germination index (GI) values below 50% were considered highly toxic, values 50%–80% and above 80% were considered low and non-toxic, respectively (Agrawal and Shahi, 2017).

Seed germination test results were reported as seed germination (*SG*%), germination index (*GI*), relative seed germination (RSG), relative root growth (RRG), and phytotoxicity (%) (Subudhi et al., 2026).

$$\text{SG}=\left(\text{No of seeds germinated on treatment}/\text{No of seeds tested}\right)\times 100$$

$$\text{RSG}=\left(\text{Number of seeds germinated on treatment}/\text{Number of seeds germinated in distilled water}\right)\times 100$$

$$\text{RRG}=\left(\text{Mean root length in treatment}/\text{Mean root length in distilled water}\right)\times 100$$

$$\text{GI}=\left(\text{RSG}\times \text{RRG}\right)/100$$

$$\text{Phytotoxicity }\%=\left[\left(\text{Radicle length of control}-\text{Radicle length of treatment}\right)/\text{Radicle length of control}\right]\times 100$$

### 2.13 Screening of petroleum hydrocarbon degradation potential

The potential of VITSVK4 to degrade benzene, toluene, xylene (BTX), phenol, mineral oil, crude oil, and used engine oil was evaluated to assess its potential to degrade a wide range of petroleum hydrocarbons. 50 mL of BH broth in a 250 mL Erlenmeyer flask with 1% (v/v) petroleum hydrocarbons and 1% inoculum (v/v) was incubated under static conditions for 7 days. Sterilized, uninoculated abiotic control was maintained in 50 mL of BH broth with the respective petroleum hydrocarbons, and incubated under the same conditions to account for abiotic and non-biological loss. After incubation, broth is centrifuged and extracted with hexane to estimate biodegradation. Biodegradation of phenol and BTX compounds was estimated by measuring absorbance at each compound’s λ max (Sumathi and Manian, 2024), determined by scanning the spectrum from 200 to 700 nm: benzene (253 nm), toluene (260 nm), xylene (261 nm) (Friedt et al., 2025), and phenol (270 nm) (Kato et al., 2021). Degradation of crude oil, mineral oil, and used engine oil was measured gravimetrically (Soumeya et al., 2022). The biodegradation with gravimetry was estimated with

$$\text{Biodegradation }\left(\%\right)=\left[\left(\text{Weight of oil in control }–\text{ Weight of oil in test}\right)\;/\text{ Weight of oil in control}\right]\times 100$$

### 2.14 Biosurfactant extraction

Cell free supernatant obtained on centrifugation for 15 min at 10000 rpm 4 °C was acidified to pH 2.0 and maintained at 4 °C overnight. After incubation, the supernatant was centrifuged, and ethyl acetate was used for extraction of organic phase. Crude biosurfactant obtained after evaporation of ethyl acetate was subjected to FTIR analysis for characterization (Gayathri et al., 2025).

### 2.15 Fourier Transform Infrared (FTIR) spectroscopy analysis

Crude biosurfactant of VITSVK4 was directly subjected to FTIR analysis to analyse its functional groups. FTIR analysis was carried out in the 400–4000 cm^-1^ range using a spectrophotometer (Agilent Cary 630, United States of America) at 4 cm^-1^ resolution with 24 scans per sample. FTIR analysis was performed in diamond ATR mode, enabling direct analysis of the extracted biosurfactant. Data was analysed and plotted with Agilent MicroLab.

### 2.16 Biosurfactant stability studies

VITSVK4 degraded broth was centrifuged, and the cell free supernatant containing biosurfactant was subjected to temperatures ranging from 30 °C to 80 °C, pH ranging from 2 to 12, and 1%–10% NaCl. pH was altered with 1 N HCl and 1 N NaOH, and salinity was adjusted with NaCl. Cell free supernatant was subjected to these conditions for 3 h, and biosurfactant stability was measured with the E24 index (Zargar et al., 2022). Residual activity was measured for 40 °C and 50 °C, pH 5 and 9, and salinity 1% and 5%, with E24 index at 30 °C representing without treatment values. Residual activity was calculated with (Raddadi et al., 2018)

$$\text{Residual activity }\%=\left(E24\text{ index after treatment }/\;E24\text{ index without treatment}\right)\;x\;100$$

### 2.17 Oil removal assay

Soil used for the oil removal assay was subjected to sterilization for 3 days at 80 °C. Soil (20 g) was mixed with 10% (v/w) of used engine oil and left in an orbital shaker for 24 h. Cell free supernatant (40 mL), Triton X-100 (6.25 µL in 40 mL distilled water), and distilled water (40 mL) was added to the soil mixed with oil and incubated for 24 h. The contents were subjected to centrifugation at 6000 rpm for 20 min, and solvent extraction was performed with hexane. Hexane was collected in a pre-weighed beaker, and the oil recovery was determined with the formula (Ali et al., 2019).

$$\text{Oil removal }\left(\%\right)=[\left(\text{Weight of oil used }–\text{ Weight of oil left in soil}\right)\;/\text{ Weight of oil used}]\times 100$$

### 2.18 Statistical analysis

All the experiments were performed in independent biological triplicates, and the values were expressed as mean ± standard deviation. In seed germination test, GraphPad Prism v.8.0.2.263 was used for one-way ANOVA, and *P* value <0.05 was considered statistically significant.

## 3 Results

### 3.1 Isolation

The BH broth supplemented with phenanthrene as the sole carbon source was used for isolating phenanthrene degrading bacteria under static conditions. Based on distinct colony morphology, two isolates designated as VITSVK4 and VITSVK5 were selected as potential phenanthrene degraders.

### 3.2 Phenanthrene biodegradation and biosurfactant screening

VITSVK4 degraded 45% ± 2.08% of 100 mg/L phenanthrene within 7 days and exhibited higher biosurfactant production compared to VITSVK5. The detailed screening results are summarized in Table 1.

**TABLE 1:** Phenanthrene degradation and biosurfactant screening of VITSVK4 and VITSVK5.

*Isolate/control*	*Phenanthrene degradation (%)*	*Oil displacement assay (cm)*	*Drop collapse assay*	*Emulsification index (%)*
VITSVK4	45 ± 2.08	5 ± 0.5	++ ve	73 ± 2.30
VITSVK5	29 ± 1.73	2.5 ± 0.6	++ ve	62 ± 2.08
Triton X-100	-	7.8 cm ± 0.3	+++ ve	-

### 3.3 Biofilm test

Biofilm formation with tube method, demonstrated visible bacterial adhesion to the surface in both isolates, indicating biofilm production. 96-well microtiter plate assay established both VITSVK4 and VITSVK5 as strong biofilm producers, as the OD of the isolates exceeded that of the negative control by four times (4×OD_C_ < OD).

### 3.4 Preliminary enzyme screening assays

VITSVK4 exhibited positive activity for both lipase and laccase assays, and VITSVK5 showed positive activity only for lipase. Based on the screening results, VITSVK4 was selected for subsequent experiments.

### 3.5 Molecular characterization of VITSVK4 with 16S rRNA sequencing

The isolate VITSVK4 was identified based on 16S rRNA sequencing. A 1418 bp sequence representing the 16S rRNA gene was amplified, sequenced, and deposited in the GenBank database under accession number PX485663. BLASTN analysis of the obtained sequence, with a query coverage of 100%, indicated that VITSVK4 shared 99.44% sequence similarity with *Stenotrophomonas maltophilia* strain IAM 12423. Based on 16S rRNA gene sequencing, VITSVK4 was tentatively identified as *Stenotrophomonas maltophilia*. The phylogenetic tree of *S. maltophilia* VITSVK4 is given in Figure 1.

**FIGURE 1:**
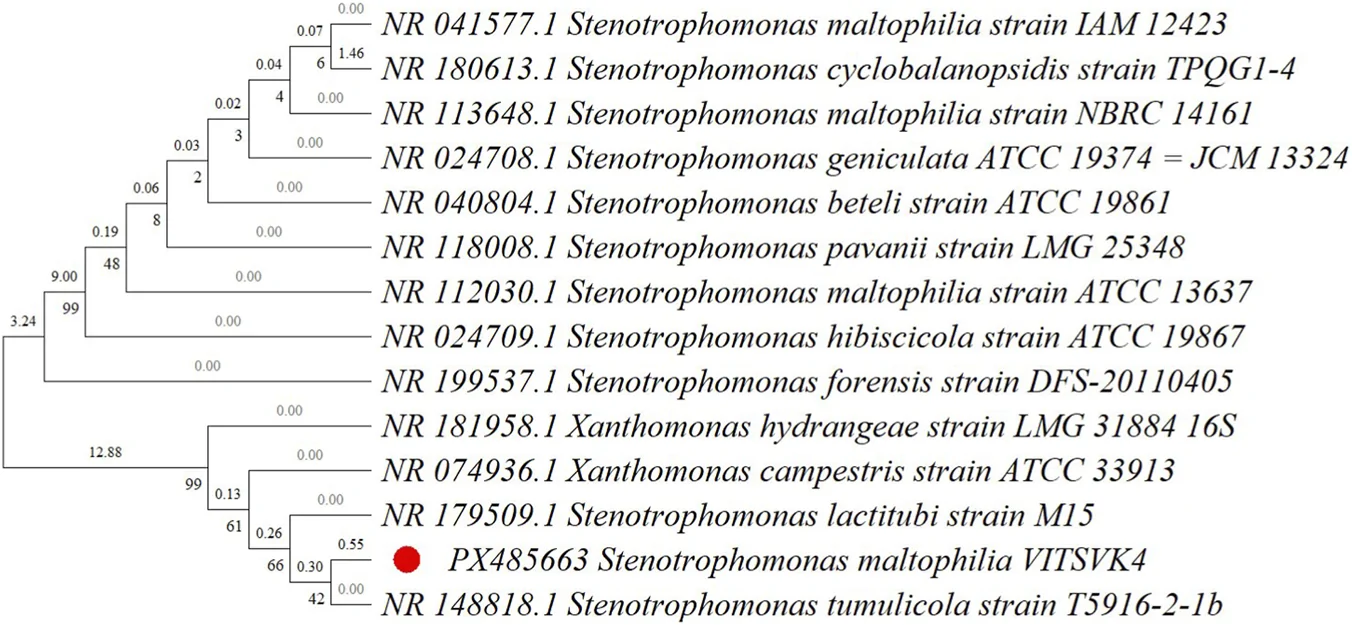
Phylogenetic tree of *Stenotrophomonas maltophilia* VITSVK4.

### 3.6 Effect of nitrogen containing compounds on phenanthrene degradation

Among the nitrogen containing compounds tested, yeast extract resulted in the highest degradation of 54% ± 1.6%, followed by soybean, while peptone exhibited the lowest degradation. Yeast extract at 0.3% (w/v) yielded the highest phenanthrene degradation (66% ± 2.3%) and was employed for growth study.

### 3.7 Estimation of growth and optimization

The growth of *S. maltophilia* with phenanthrene and 0.3% yeast extract under non agiatated, static conditions was quantified from Day 1–10 by measuring dried biomass. Dried biomass weight increased progressively until Day 8, reaching a maximum of 0.74 ± 0.05 g/L, followed by a decline in weight on subsequent days. OFAT screening identified 32 °C, pH 7, an inoculum volume of 2% (v/v), and 8 days as the best performing conditions among the tested levels, as the highest degradation was observed. Under these conditions, a maximum degradation of 72% ± 1.73% was achieved at 100 mg/L phenanthrene. A gradual decrease in degradation was observed with increasing phenanthrene concentration, with 59.3% ± 2.08% degradation at 500 mg/L under non-agitated conditions, and a pronounced decline in degradation was observed from 600 mg/L. These findings highlight the importance of nutrients and environmental parameters in enhancing biodegradation performance.

### 3.8 Gas chromatography mass spectrometry

GC-MS analysis was performed to validate phenanthrene degradation. The chromatogram of the control showed a single prominent peak at retention time 15.20, corresponding to phenanthrene. The chromatogram of degraded phenanthrene showed no detectable peak at the phenanthrene retention time, consistent with the removal of the parent compound. Hexane, used for solvent extraction is not suited to recover the polar, acidic transformation products of phenanthrene. These results support the degradation of phenanthrene by *S. maltophilia* VITSVK4 under static conditions. GC-MS chromatograms of phenanthrene before and after biodegradation are given in Figure 2.

**FIGURE 2:**
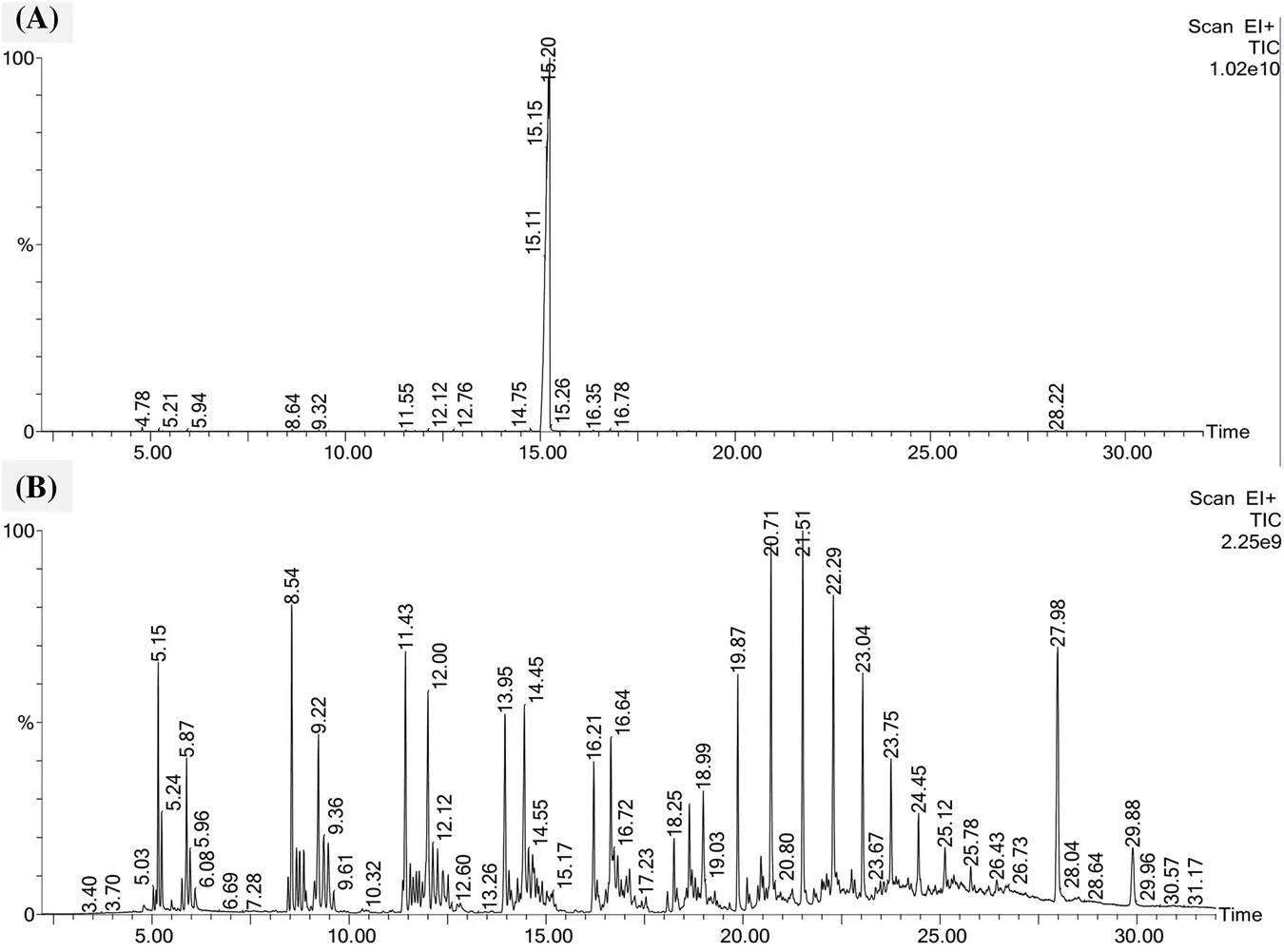
GC-MS chromatogram of **(A)** phenanthrene (control) and **(B)** VITSVK4 biodegraded phenanthrene.

### 3.9 Seed germination test

The phytotoxicity of VITSVK4 degraded phenanthrene and untreated phenanthrene was assessed with *Vigna radiata* and *Triticum aestivum*. In *V. radiata,* seed germination was 100% in distilled water, 90% in treated phenanthrene, and 10% in untreated phenanthrene. Similarly, in *T. aestivum*, 90% and 70% germination were observed in distilled water and treated phenanthrene, respectively, while untreated phenanthrene showed no germination. Shoot and root length of *V. radiata* and *T*. *aestivum* under different treatments are given in Figure 3. In *V. radiata*, phytotoxicity (%) for treated and untreated phenanthrene was 24.5% and 94.7%, respectively. VITSVK4 treated phenanthrene showed 13.1% phytotoxicity on *T*. *aestivum*. The germination index for *V. radiata* calculated from RSG and RRG was 67.5% and 0.5% for treated and untreated phenanthrene, respectively, and it was 66.8% for treated phenanthrene on *T*. *aestivum*. The GI values of both plant species indicate low toxicity with treated phenanthrene and significant toxicity with untreated phenanthrene, indicating reduced phytotoxicity after biodegradation. Untreated phenanthrene exhibited significant reduction in growth compared to treated and distilled water. For *V. radiata* root length, *T*. *aestivum* shoot and root length, treated was not significantly different from distilled water. *V. radiata* shoot length showed effects of partial toxicity, with growth in phenanthrene treated seeds significantly lower than distilled water.

**FIGURE 3:**
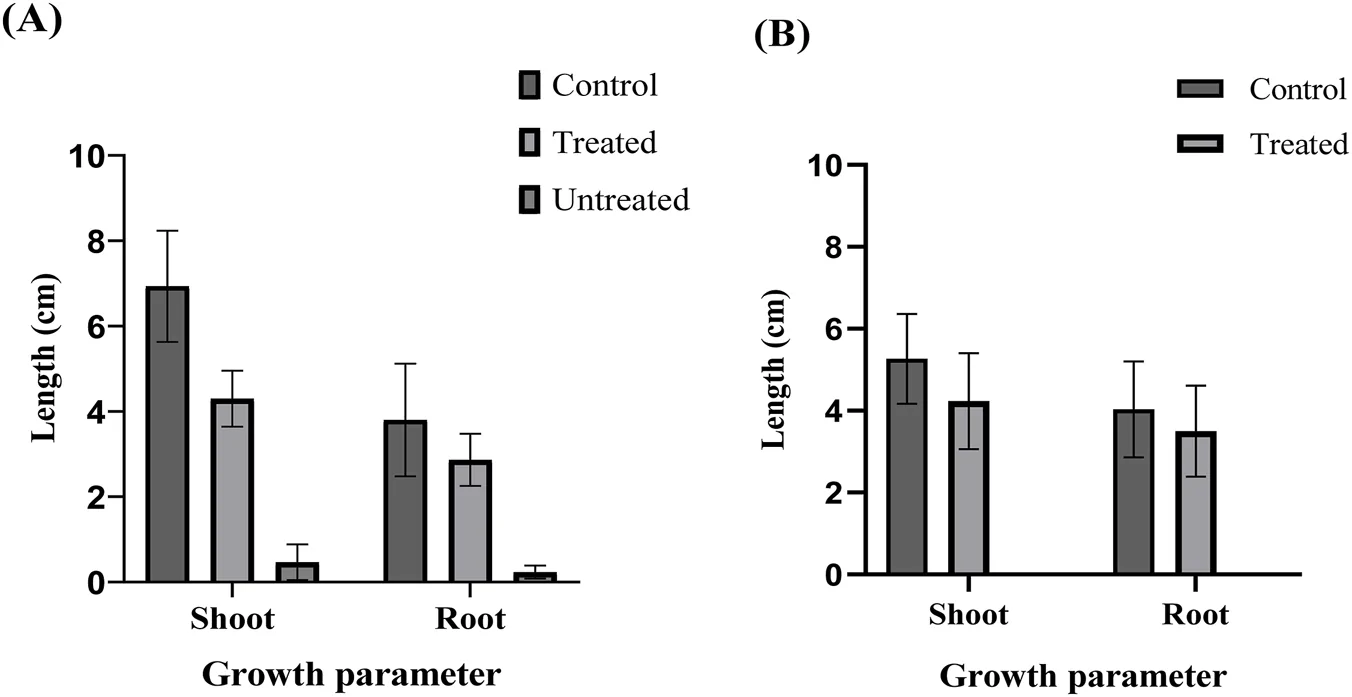
Effects of treated and untreated phenanthrene on shoot and root elongation of **(A)** *Vigna radiata* and **(B)** *Triticum aestivum*.

Statistical analysis was performed using one way ANOVA followed by Tukey’s *post hoc* test for three biological replicates and *P* value <0.05 was considered statistically significant. For, *V. radiata* shoot length, normality of residuals was confirmed by Shapiro-Wilk (*P* = 0.9384), and homogeneity was confirmed by Brown-Forsythe test (*P* = 0.4094). One way ANOVA showed significant variation among groups (F (2,6) = 4.144, *P* = 0.0003, R^2^ = 0.9325). Tukey’s *post hoc* test confirmed significant difference across all pairs: control to treated (*P* = 0.0239), control vs. untreated (*P* = 0.0003), and treated vs. untreated (*P* = 0.0041). For, *V. radiata* root length, normality of residuals was confirmed by Shapiro-Wilk (*P* = 0.6767), and homogeneity was confirmed by Brown-Forsythe test (*P* = 0.4295). One way ANOVA showed significant variation among groups (F (2,6) = 14.34, *P* = 0.0052, R^2^ = 0.8270). Tukey’s *post hoc* test confirmed that untreated had significant reduction compared to control (*P* = 0.0050) and treated (*P* = 0.0207) and root length in treated group did not differ significantly from control (*P* = 0.4209).

For *T. aestivum* shoot length, normality of residuals was confirmed by Shapiro-Wilk (*P* = 0.9116), and homogeneity was confirmed by Brown-Forsythe test (*P* = 0.4259). One way ANOVA showed significant variation among groups (F (2,6) = 27.20, *P* = 0.0010, R^2^ = 0.9007). Tukey’s *post hoc* test confirmed that untreated had significant reduction compared to control (*P* = 0.0011) and treated (*P* = 0.0034) and shoot length in treated group did not differ significantly from control (*P* = 0.4143). For *T. aestivum* root length, normality of residuals was confirmed by Shapiro-Wilk (*P* = 0.1936), and homogeneity was confirmed by Brown-Forsythe test (*P* = 0.3741). One way ANOVA showed significant variation among groups (F (2,6) = 16.53, *P* = 0.0036, R^2^ = 0.8464). Tukey’s *post hoc* test confirmed that untreated had significant reduction compared to control (*P* = 0.0044) and treated (*P* = 0.0089) and root length in treated group did not differ significantly from control (*P* = 0.7724).

### 3.10 Screening of petroleum hydrocarbon degradation potential

*Stenotrophomonas maltophilia* VITSVK4 demonstrated degradation of monoaromatic hydrocarbons including benzene, toluene, and xylene as well as complex petroleum hydrocarbon mixtures such as mineral oil, used engine oil, and crude oil. The highest degradation was observed in benzene, followed by toluene, while phenol showed the lowest degradation efficiency. Screening of the degradation potential of *S. maltophilia* VITSVK4 with various petroleum hydrocarbons is shown in Figure 4.

**FIGURE 4:**
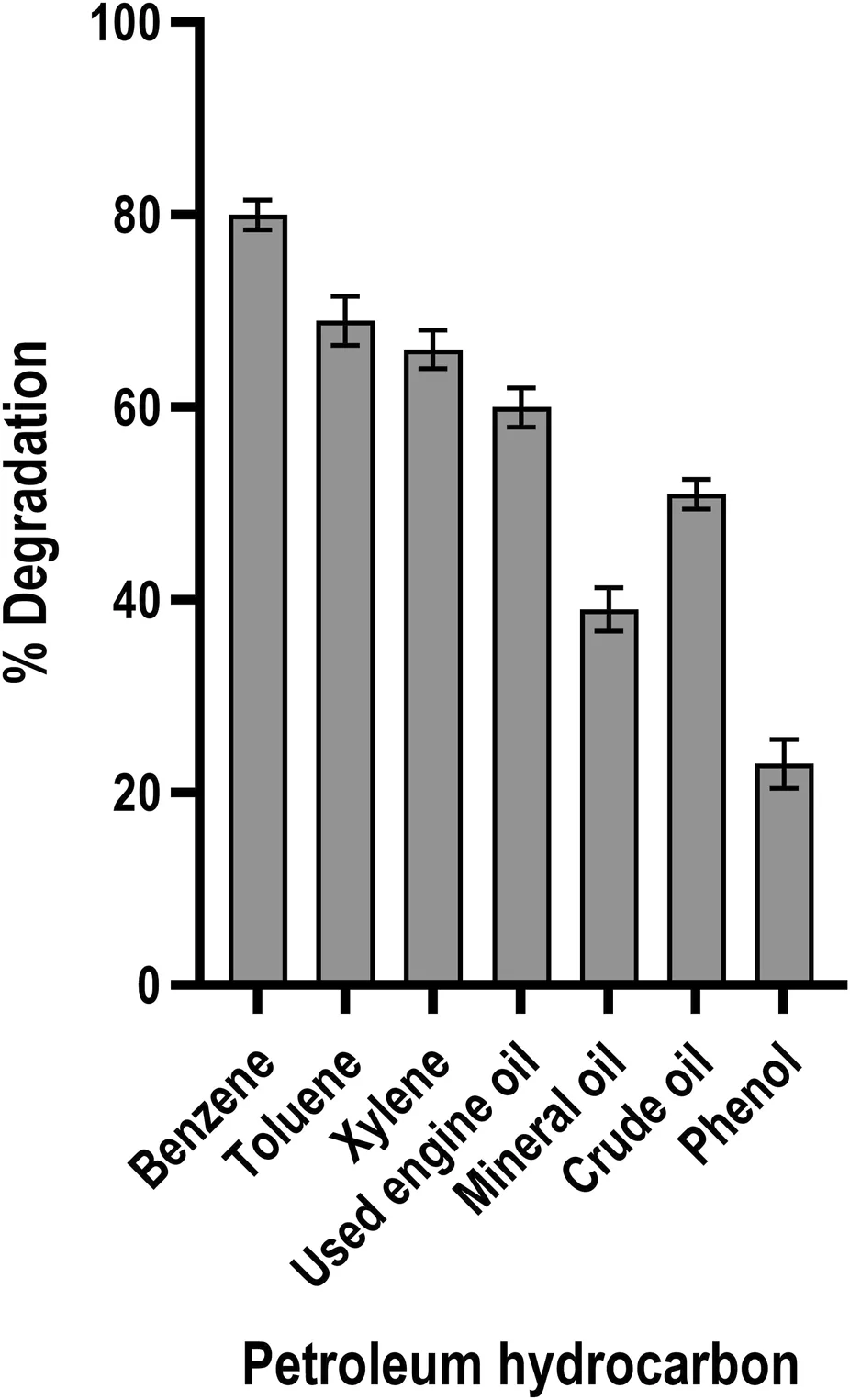
Screening of the degradation potential of *S. maltophilia* VITSVK4 with various petroleum hydrocarbons.

### 3.11 Fourier Transform Infrared (FTIR) spectroscopy analysis

The crude biosurfactant produced by VITSVK4 was characterized using FTIR to identify its functional groups. A prominent peak at 2984 cm^-1^ corresponds to C-H stretching vibrations, suggesting aliphatic lipid chains, while the peak at 1736 cm^-1^ corresponds to C=O stretching, indicating ester linkages and a lipid component. The peaks observed at 1233 cm^-1^, 1097 cm^-1,^ and 1043 cm^-1^ correspond to C–O–C and C–O stretching vibrations, indicating the presence of sugar moieties. The coexistence of both lipid and sugar components suggests a glycolipid type biosurfactant. The FTIR spectrum of crude biosurfactant is given in Figure 5.

**FIGURE 5:**
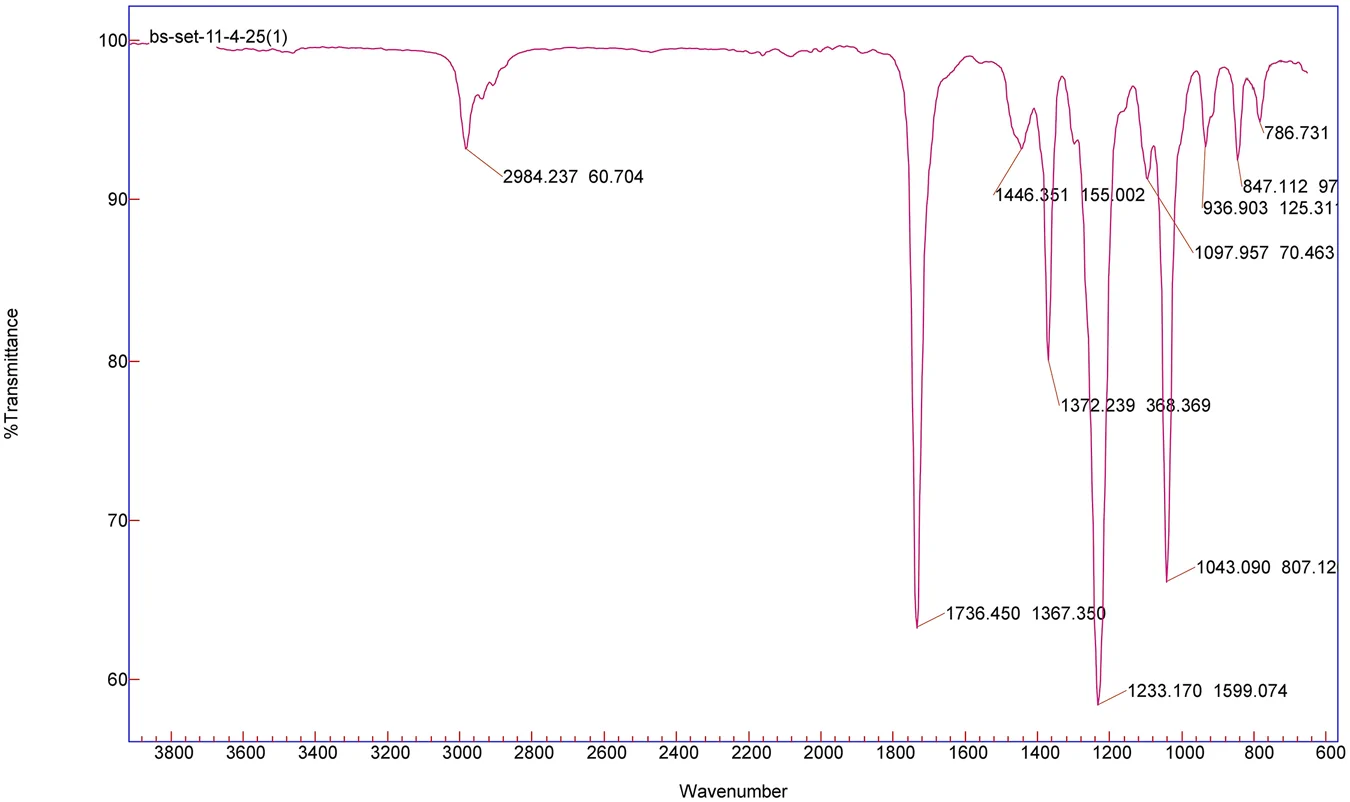
FTIR spectrum of VITSVK4 crude biosurfactant.

### 3.12 Biosurfactant stability

The stability of the biosurfactant was evaluated under a range of temperature, pH, and salinity. VITSVK4 biosurfactant was stable around pH 5 to 9, temperature up to 50 °C, and salinity up to 5%, beyond which decline in activity was observed. These results demonstrate that the biosurfactant produced by VITSVK4 exhibits environmental stability, and the E24 index under different treatments is given in Figure 6. Residual activity at 40 °C and 50 °C was 94.9% ± 2.73%, and 84.3% ± 2.59% respectively. At, pH 5 and 9, 83.9% ± 2.59% and 81.8% ± 21.6% residual activity was observed respectively. Residual activity at 1% salinity was 83.5% ± 2.04% and 67.4% ± 2.59% for 5% salinity.

**FIGURE 6:**
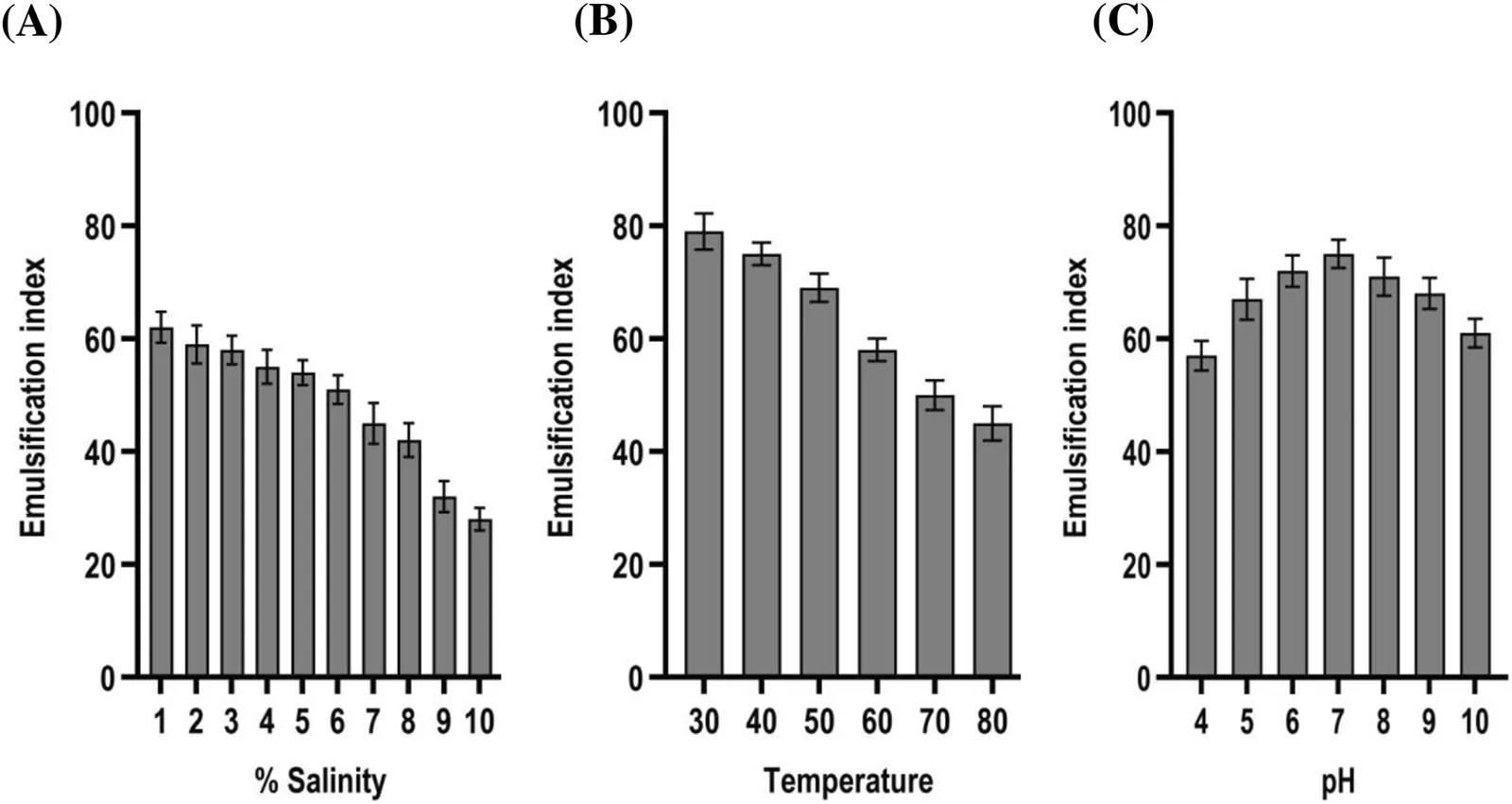
E24 index of VITSVK4 biosurfactant under various **(A)** pH **(B)** temperature, and **(C)** salinity.

### 3.13 Oil removal assay

The oil removal assay evaluated the efficiency of the biosurfactant in recovering residual oil. The extracted biosurfactant recovered 60% ± 2.51% oil, compared to 19% ± 3.05% with distilled water, while Triton X-100 recovered 73% ± 2.08% oil, highlighting the potential of *S. maltophilia* in oil recovery.

## 4 Discussion

This study demonstrates phenanthrene degradation and biosurfactant production under static conditions by *Stenotrophomonas maltophilia* VITSVK4. The combined characteristics of phenanthrene degradation, biosurfactant production, and biofilm formation under static, non-agitated conditions highlight its potential as a promising and effective candidate for bioremediation. *S. maltophilia* VITSVK4 was isolated from uncontaminated terrestrial soil as it hosts a rich and largely untapped microbial community. Since pristine terrestrial soil has a vast microbial diversity, it is better for identifying novel and effective biodegraders (Labbé et al., 2007). Isolation of novel, adaptable microbes with the potential for bioremediation can be enhanced by focusing on such unexplored environments.

Majority of contaminated sites, including subsurface soil and aquatic sediments, are characterized by limited oxygen, which restricts the activity of strictly aerobic microbes. Bacteria adapted to limited oxygen conditions, such as microaerophilic degraders, are advantageous as they have the potential to degrade petroleum hydrocarbons under such conditions (Rather et al., 2025). Li et al. (2017) isolated bacteria capable of degrading phenanthrene and pyrene under hypoxic conditions, demonstrating the adaptability of PAH degraders to oxygen limited environments. *Pseudomonas stutzeri* JP1, capable of degrading phenanthrene under low oxygen conditions, was reported by Kong et al. (2017). A phenanthrene degrading, microbial consortium W4 exhibited significant degradation of phenanthrene under static conditions (Wang et al., 2007). Similarly, the present study isolated two phenanthrene degrading bacterial isolates, capable of degradation under static conditions. These findings indicate that microorganisms can effectively degrade PAHs under static conditions, making them promising candidates for the bioremediation of contaminated soils and sediments.

*S. maltophilia* VITSVK4 was screened for biosurfactant production and biofilm formation, key functional traits for effective petroleum hydrocarbon biodegradation. Biosurfactants reduce surface tension and enhance solubilization of hydrophobic PAHs, thus increasing their bioavailability and biodegradation. Rhamnolipid biosurfactant was reported to increase phenanthrene biodegradation in an aqueous system (Zhao et al., 2011). Degradation of multiple PAHs present in crude oil by a co culture was reported to improve by 10% with the addition of rhamnolipid biosurfactant (Kumari et al., 2018). Biofilm formation creates a protective matrix that concentrates cells at the pollutant interface, provides tolerance against toxic chemicals and varying environmental conditions, and retains enzymes to achieve higher degradation rates than planktonic cells (Mishra et al., 2022). Biofilm formation promotes PAH biodegradation as it enhances bioavailability, improving biodegradation. PAH degradation facilitated by biofilms of multiple bacteria, including *Stenotrophomonas acidaminiphila* NCW-702, exhibited higher degradation than planktonic cells (Mangwani et al., 2016). Bacteria with the ability to produce biosurfactant and form biofilms were reported to effectively degrade petroleum hydrocarbon mixtures and cyclic hydrocarbons (Meliani and Bensoltane, 2014). *Stenotrophomonas maltophilia* VITSVK4, a biosurfactant producer with strong biofilm formation under static conditions, is a potential candidate for bioremediation of petroleum hydrocarbons.

Nitrogen source is essential for bacterial growth and directly impacts microbial metabolism and petroleum hydrocarbon degradation efficiency in contaminated soils (Ou et al., 2025). Among the tested nitrogen containing compounds, *S. maltophilia* VITSVK4 exhibited the highest degradation with yeast extract, as it is rich in amino acids, nucleic acids, vitamins, minerals, and polypeptides, accelerating microbial metabolism and growth (Tao et al., 2023). Similarly, crude oil degradation as well as biosurfactant production by *Acinetobacter baumannii* OCB increased with supplementation with yeast extract (Goveas and Sajankila, 2020). Optimization studies revealed that time, pH, temperature, and inoculum significantly influence its ability to degrade phenanthrene. *Stenotrophomonas maltophilia* VITSVK4 showed a steady increase in growth, reaching its highest growth on day 8 under non-agitated conditions, and the highest phenanthrene degradation occurred at pH 7 and 32 °C. Arulazhagan et al. (2017) reported that the acidophilic *Stenotrophomonas maltophilia* AJH1 degraded 50 mg/L phenanthrene at 30 °C, and addition of yeast extract enhanced degradation. Similarly, *Alcaligenes ammonioxydans* VITRPS2 reached 72% phenanthrene removal in 7 days with yeast extract, pH 8, 37 °C, and an 8% inoculum (Sumathi and Manian, 2024). *Rhodococcus* sp. exhibited phenanthrene (70 mg/L) degradation at 30 °C and pH 7 (Bishnoi et al., 2024).

Beyond phenanthrene, *S. maltophilia* VITSVK4 showed the potential to degrade mineral oil, phenol, crude oil, and monoaromatics toluene, benzene, and xylene. Isolates from the genus *Rhodococcus*, *Pseudomonas,* and *Mycobacterium* were reported to degrade a wide range of pollutants, including n-alkanes and PAHs, and to exhibit degradation under various environmental conditions (Brzeszcz and Kaszycki, 2018). *Pseudomonas aeruginosa* AQNU-1 exhibited degradation of n-alkanes, alkylcyclohexane, alkylbenzene, alkyltoluene, and aromatic compounds in crude oil under non-agitated conditions (Liu et al., 2022). *Pseudomonas maltophilia* was reported to degrade toluene and xylene under low oxygen conditions, but did not exhibit benzene degradation (Su and Kafkewitz, 1994), and *Stenotrophomonas maltophilia*-SR1 exhibited benzene, toluene, and xylene degradation under agitated conditions (Bashandy et al., 2020). *S. maltophilia* SVIA2 exhibited potential to degrade PAHs, and the genome was reported to contain essential genes required for PAH degradation (Elufisan et al., 2019). The broad metabolic range of VITSVK4 highlights its potential to remediate complex petroleum hydrocarbon pollution.

FTIR analysis of VITSVK4 crude biosurfactant displayed the characteristic functional groups, indicating a glycolipid type biosurfactant with lipid and carbohydrate components. These features are similar to a report on rhamnolipid biosurfactants produced by *Stenotrophomonas maltophilia NBS-11*, where peaks representing polysaccharides and lipids were detected (Hemlata et al., 2015). FTIR spectrum of *S. maltophilia* IITR87 also showed similar functional groups, indicating it to be a rhamnolipid (Tripathi et al., 2020), supporting the characterization of VITSVK4 biosurfactant as a glycolipid-type biosurfactant.

The biosurfactant extracted from VITSVK4 exhibited stability under pH (5–9), temperatures up to 50 °C, and 5% NaCl. Similarly, the biosurfactant produced by *Gordonia* sp. IITR100 was stable at pH 6 to 10, temperature up to 30 °C, and 2% NaCl (Zargar et al., 2022). The stability range exhibited by VITSVK4 was narrower than that of *Stenotrophomonas nematodicola* W5, which remained stable up to 120 °C and 7.5% NaCl (Jiang et al., 2024). *S. maltophilia* IITR87 exhibited stability from pH 2 to 12, up to 120 °C, and 14% NaCl, but the highest E24 index was around 53% (Tripathi et al., 2020), whereas VITSVK4 exhibited the highest E24 index of 79.2% ± 2.51% at 30 °C. These observations suggest that the biosurfactant produced by VITSVK4 shows moderate tolerance to environmental variability. In our study, the biosurfactant achieved an oil recovery of approximately 60% ± 2.5%, indicating potential for oil recovery applications. In static oil washing experiment, *Stenotrophomonas nematodicola* W5 achieved 7.1% oil recovery, while *Bacillus paralicheniformis* LXJ3 and *Pseudomonas aeruginosa* DSM50071 exhibited 23.4% and 28.1% oil recovery, respectively (Jiang et al., 2024). The enhanced recovery observed in VITSVK4 highlights its potential as an effective agent for oil recovery. These results establish *Stenotrophomonas maltophilia* VITSVK4 as an efficient phenanthrene degrader under static conditions.

*Stenotrophomonas maltophilia* is a recognized opportunistic human pathogen. While the species is ubiquitous in environment and VITSVK4 was isolated from uncontaminated terrestrial soil with no clinical association, the opportunistic pathogenicity means that any environmental application would require rigorous biosafety evaluation. An alternative use would be to use cell free extracts, purified enzymes, or biosurfactant, rather than live organism for petroleum hydrocarbon bioremediation. Such applications would require comprehensive characterization of biosurfactant and enzymes, which was not performed in this study and acknowledged as limitations of this study. The present work was conducted entirely under laboratory conditions and did not assess field performance, environmental persistence, ecological compatibility, which is important to determine the safety and efficiency of bioremediation. Biosafety assessment, characterization of enzymes and biosurfactant are identified as the future directions before any considerations of real world remediation strategies.

## 5 Conclusion

*Stenotrophomonas maltophilia* VITSVK4 demonstrated efficient phenanthrene degradation under static conditions, along with biosurfactant production. GC-MS analysis confirmed phenanthrene degradation, supporting its potential as a phenanthrene degrading isolate. The isolate also showed degradation potential against aliphatic and aromatic compounds, demonstrating a broad substrate range. FTIR analysis indicated a glycolipid type biosurfactant, stable under varying pH, temperature, and salinity. Collectively, these findings highlight the potential of VITSVK4 as a promising candidate for phenanthrene bioremediation under static, non-agitated conditions.

## Data Availability

The datasets presented in this study can be found in online repositories. The names of the repository/repositories and accession number(s) can be found below: https://www.ncbi.nlm.nih.gov/genbank/, PX485663.

## References

[B1] Agrawal N. Shahi S. K. (2017). Degradation of polycyclic aromatic hydrocarbon (pyrene) using novel fungal strain *Coriolopsis byrsina* strain APC5. Int. Biodeterior. Biodegr. 122, 69–81. 10.1016/j.ibiod.2017.04.024

[B2] Ali N. Wang F. Xu B. Safdar B. Ullah A. Naveed M. (2019). Production and application of biosurfactant produced by *Bacillus licheniformis* Ali5 in enhanced oil recovery and motor oil removal from contaminated sand. Molecules 24 (24), 4448. 10.3390/molecules24244448 31817293 PMC6943484

[B3] Arulazhagan P. Al-Shekri K. Huda Q. Godon J. J. Basahi J. M. Jeyakumar D. (2017). Biodegradation of polycyclic aromatic hydrocarbons by an acidophilic *Stenotrophomonas maltophilia* strain AJH1 isolated from a mineral mining site in Saudi Arabia. Extremophiles 21, 163–174. 10.1007/s00792-016-0892-0 27848015

[B4] Bashandy S. R. Abd-Alla M. H. Dawood M. F. (2020). Alleviation of the toxicity of oily wastewater to canola plants by the N2-fixing, aromatic hydrocarbon biodegrading bacterium *stenotrophomonas maltophilia*-SR1. Appl. Soil Ecol. 154, 103654. 10.1016/j.apsoil.2020.103654

[B5] Bishnoi K. Rani P. Bishnoi N. (2024). Optimization of phenanthrene biodegradation process by isolated bacterial strain *rhodococcus* sp. using response surface methodology. Res. J. Chem. Environ. 28 (7), 15–26. 10.25303/287rjce015026

[B6] Brzeszcz J. Kaszycki P. (2018). Aerobic bacteria degrading both *n*-alkanes and aromatic hydrocarbons: an undervalued strategy for metabolic diversity and flexibility. Biodegradation 29, 359–407. 10.1007/s10532-018-9837-x 29948519

[B7] Chowdhury R. Dhar K. Ahamed F. Alam S. Uddin M. (2017). Biodegradation of anthracene and phenanthrene by bacteria isolated from oil-contaminated soil of Bangladesh. Chem. Ecol. 33 (9), 843–855. 10.1080/02757540.2017.1382481

[B8] Dhanasekaran A. Tiwari K. Bhange A. Ghosh S. S. Kannabiran K. (2025). Biodegradation of expanded polystyrene using *Pseudomonas aeruginosa* VITARK5. Detritus 30, 99–108. 10.31025/2611-4135/2025.19475

[B9] Duarte H. Aliaño-González M. J. Romano A. Medronho B. (2024). Advancements in detection and mitigation strategies for petroleum-derived contaminants in aquatic environments: a comprehensive review. Sensors 24 (11), 3284. 10.3390/s24113284 PMC11174374 38894076

[B10] Elufisan T. O. Lozano L. Bustos P. Rodríguez-Luna I. C. Sánchez-Varela A. Oyedara O. O. (2019). Complete genome sequence of *Stenotrophomonas maltophilia* strain SVIA2, isolated from crude oil-contaminated soil in Tabasco, Mexico. Microbiol. Resour. Announc. 8 (30), e00529–19. 10.1128/mra.00529-19 31346014 PMC6658684

[B11] Friedt J. M. Luzet V. Soumann V. Nief N. Segues B. Pucheu G. (2025). Specific detection of BTEX contamination in water using a π-Hole-Catching surface acoustic wave sensor. ACS Omega 10 (11), 10903–10910. 10.1021/acsomega.4c08826 PMC11947771 40160748

[B12] Gayathri H. Yuvaraj S. Kannabiran K. (2025). Petroleum hydrocarbon degradation potential of biosurfactant extracted from bacteria isolated from oil contaminated sites. Res. J. Chem. Environ. 29 (12), 14–22. 10.25303/2912rjce014022

[B13] Goveas L. C. Sajankila S. P. (2020). Effect of yeast extract supplementation on halotolerant biosurfactant production kinetics coupled with degradation of petroleum crude oil by *Acinetobacter baumannii* OCB1 in marine environment. Bioresour. Technol. Rep. 11, 100447. 10.1016/j.biteb.2020.100447

[B14] Harika K. Shenoy V. P. Narasimhaswamy N. Chawla K. (2020). Detection of biofilm production and its impact on antibiotic resistance profile of bacterial isolates from chronic wound infections. J. Glob. Infect. Dis. 12 (3), 129–134. 10.4103/jgid.jgid_150_19 PMC7733422 33343163

[B15] Hemlata B. Selvin J. Tukaram K. (2015). Optimization of iron chelating biosurfactant production by *Stenotrophomonas maltophilia* NBS-11. Biocatal. Agric. Biotechnol. 4 (2), 135–143. 10.1016/j.bcab.2015.02.002

[B16] Imam A. Kanaujia P. K. Ray A. Suman S. K. (2021). Removal of petroleum contaminants through bioremediation with integrated concepts of resource recovery: a review. Indian. J. Microbiol. 61, 250–261. 10.1007/s12088-021-00928-4 PMC8263831 34294990

[B17] Jiang M. Wang H. Liu J. Hou X. Zhang Y. Liu X. (2024). Isolation and characterization of biosurfactant-producing bacteria for enhancing oil recovery. Processes 12 (11), 2575. 10.3390/pr12112575

[B18] Juhasz A. L. Stanley G. A. Britz M. L. (2000). Microbial degradation and detoxification of high molecular weight polycyclic aromatic hydrocarbons by *Stenotrophomonas maltophilia* strain VUN 10,003. Lett. Appl. Microbiol. 30 (5), 396–401. 10.1046/j.1472-765x.2000.00733.x 10792670

[B19] Kato S. Sakai Y. Sato Y. Kansha Y. (2021). The effect of high-frequency ultrasound on the photocatalytic decomposition of organic compounds in water. Chem. Eng. Trans. 88, 379–384. 10.3303/CET2188063

[B20] Kong J. Wang H. Liang L. Li L. Xiong G. Hu Z. (2017). Phenanthrene degradation by the bacterium *Pseudomonas stutzeri* JP1 under low oxygen condition. Int. Biodeterior. Biodegr. 123, 121–126. 10.1016/j.ibiod.2017.06.001

[B21] Kumari S. Regar R. K. Manickam N. (2018). Improved polycyclic aromatic hydrocarbon degradation in a crude oil by individual and a consortium of bacteria. Bioresour. Technol. 254, 174–179. 10.1016/j.biortech.2018.01.075 29413920

[B22] Kumari B. Chandra H. Chandra R. (2022). Detection of pyrene degrading bacterial strains (LOP-9 *Staphylococcus aureus* and GWP-2 *mycobacterium Vaanbaalenii*) and their metabolic products. Clean. Chem. Eng. 4, 100080. 10.1016/j.clce.2022.100080

[B23] Labbé D. Margesin R. Schinner F. Whyte L. G. Greer C. W. (2007). Comparative phylogenetic analysis of microbial communities in pristine and hydrocarbon-contaminated alpine soils. FEMS. Microbiol. Ecol. 59 (2), 466–475. 10.1111/j.1574-6941.2006.00250.x 17313586

[B24] Lara-Moreno A. Morillo E. Merchán F. Villaverde J. (2021). A comprehensive feasibility study of effectiveness and environmental impact of PAH bioremediation using an Indigenous microbial degrader consortium and a novel strain *Stenotrophomonas maltophilia* CPHE1 isolated from an industrial polluted soil. J. Environ. Manage. 289, 112512. 10.1016/j.jenvman.2021.112512 33848881

[B25] Li C. H. Ye C. Hou X. P. Chen M. H. Zheng X. Y. Cai X. Y. (2017). Isolation and characterization of polycyclic aromatic hydrocarbon-degrading bacteria with tolerance to hypoxic environments. J. Environ. Sci. Health. A. 52 (7), 581–589. 10.1080/10934529.2017.1293991 28281889

[B26] Li S. Liu J. Fang P. (2025). Biodegradation of phenanthrene by *mycobacterium* sp. TJFP1: genetic basis and environmental validation. Microorganisms 13 (5), 1171. 10.3390/microorganisms13051171 PMC12114333 40431342

[B27] Liu H. Yang G. Jia H. Sun B. (2022). Crude oil degradation by a novel strain *Pseudomonas aeruginosa* AQNU-1 isolated from an oil-contaminated Lake wetland. Processes 10 (2), 307. 10.3390/pr10020307

[B28] Ma S. Wu T. Zhang H. Fang H. Kang J. Zhao J. (2025). A highly potent benzene-degrading bacterial strain from petroleum-contaminated soils: isolation and characterization. Bioremediation. J. 30, 1–12. 10.1080/10889868.2025.2504151

[B29] Mahmood M. H. Zhi Y. Thanoon R. D. Makky E. Rahim M. H. A. (2017). “Bioremediation of disposed engine oil for lipase production,” in FGIC 1st Conference on Governance and Integrity, 337–354.

[B30] Mandalenaki A. Kalogerakis N. Antoniou E. (2021). Production of high purity biosurfactants using heavy oil residues as carbon source. Energies 14 (12), 3557. 10.3390/en14123557

[B31] Mangwani N. Shukla S. K. Kumari S. Das S. Rao T. S. (2016). Effect of biofilm parameters and extracellular polymeric substance composition on polycyclic aromatic hydrocarbon degradation. RSC Adv. 6 (62), 57540–57551. 10.1039/C6RA12824F

[B32] Markam S. S. Raj A. Kumar A. Khan M. L. (2024). Microbial biosurfactants: green alternatives and sustainable solution for augmenting pesticide remediation and management of organic waste. Curr. Res. Microb. Sci. 7, 100266. 10.1016/j.crmicr.2024.100266 PMC11385824 39257939

[B33] Mekonnen B. A. Aragaw T. A. Genet M. B. (2024). Bioremediation of petroleum hydrocarbon contaminated soil: a review on principles, degradation mechanisms, and advancements. Front. Environ. Sci. 12, 1354422. 10.3389/fenvs.2024.1354422

[B34] Meliani A. Bensoltane A. (2014). Enhancement of hydrocarbons degradation by use of *pseudomonas* biosurfactants and biofilms. J. Pet. Environ. Biotechnol. 5 (1), 168. 10.4172/2157-7463.1000168

[B35] Mishra S. Huang Y. Li J. Wu X. Zhou Z. Lei Q. (2022). Biofilm-mediated bioremediation is a powerful tool for the removal of environmental pollutants. Chemosphere 294, 133609. 10.1016/j.chemosphere.2022.133609 35051518

[B36] Mukherjee P. Roy P. (2016). Genomic potential of *Stenotrophomonas maltophilia* in bioremediation with an assessment of its multifaceted role in our environment. Front. Microbiol. 7, 967. 10.3389/fmicb.2016.00967 27446008 PMC4916776

[B37] Nzila A. Sankara S. Al-Momani M. Musa M. M. (2018). Isolation and characterisation of bacteria degrading polycyclic aromatic hydrocarbons: phenanthrene and anthracene. Arch. Environ. Prot. 44 (2), 43–54. 10.24425/119693

[B38] Ortiz-Lechuga E. G. Almaguer-Cantú V. Herrera-Barquín H. Solís-Arévalo K. K. Batista-García R. A. Arévalo-Niño K. (2026). Production of surface-active metabolites by *bacillus* sp. from vegetable oil-impacted soil: ecological implications and screening limitations. Microbiol. Res. 17 (4), 76. 10.3390/microbiolres17040076

[B39] Ou Y. Zhang C. Wu M. Yu Y. Zhang X. Hu M. (2025). Differential roles of nitrogen and phosphorus in petroleum hydrocarbon biodegradation: metatranscriptomic insights from soil bioremediation. J. Environ. Chem. Eng. 13 (5), 118412. 10.1016/j.jece.2025.118412

[B40] Raddadi N. Giacomucci L. Marasco R. Daffonchio D. Cherif A. Fava F. (2018). Bacterial polyextremotolerant bioemulsifiers from arid soils improve water retention capacity and humidity uptake in sandy soil. Microb. Cell. Fact. 17, 83. 10.1186/s12934-018-0934-7 29855369 PMC5984429

[B41] Raheema R. Hadi S. Dirwal A. (2026). Microbial bioremediation for sustainable development. Microbe 11, 100699. 10.1016/j.microb.2026.100699

[B42] Rather M. A. Bharadwaj R. Haldar M. Sengar D. S. Verma H. G. Goswami P. K. (2025). Biodegradation of crude oil by biosurfactant-producing microaerophilic bacterium *Pseudomonas aeruginosa* MAR1. Sci. Total. Environ. 991, 179876. 10.1016/j.scitotenv.2025.179876 40543196

[B43] Sakthishabarish K. Mahesh P. Kannabiran K. (2026). Biodegradation of remazol brilliant blue R dye with a new strain of *stutzerimonas Stutzeri* VITKSS2. Appl. Biochem. Microbiol. 62, 514–525. 10.1134/S0003683825603117

[B44] Shojaei S. Mulligan C. N. (2026). Characterization of the biosurfactant produced by Indigenous bacteria from mature fine tailings. Bioengineering 13 (4), 452. 10.3390/bioengineering13040452 PMC13113356 42072246

[B45] Shokranian S. Yousefzadi M. Biuki N. A. Yaghoubi-Avini M. TaheriAkerdi M. (2025). Isolation, characterization, and bioremediation potential of oil-degrading bacteria from contaminated soils in shiraz and Bandar abbas, Iran. Sci. Rep. 15, 42101. 10.1038/s41598-025-26242-3 41298700 PMC12658252

[B46] Soumeya S. Allaoueddine B. Hocine A. K. (2022). Biodegradation of used motor oil by *Streptomyces ginkgonis* KM-1–2, isolated from soil polluted by waste oils in the region of azzaba (Skikda-Algeria). J. Biotech. 349, 1–11. 10.1016/j.jbiotec.2022.03.006 35318108

[B47] Su J. Kafkewitz D. (1994). Utilization of toluene and xylenes by a nitrate-reducing strain of *Pseudomonas maltophilia* under low oxygen and anoxic conditions. FEMS. Microbiol. Ecol. 15 (3-4), 249–257. 10.1111/j.1574-6941.1994.tb00248.x

[B48] Subudhi L. Panda A. K. Mohapatra S. (2026). Phytotoxic effects of methylene blue dye on mustard (*Brassica juncea* L.): insights from germination bioassays and multivariate analysis. Rsc. Adv. 16 (30), 27518–27530. 10.1039/d6ra02447e 42181732 PMC13195417

[B49] Sumathi K. Manian R. (2024). Degradation and inhibition kinetics of phenanthrene by *Alcaligenes ammonioxydans*, [VITRPS2] strain isolated from petroleum-contaminated soil. Discov. Appl. Sci. 6, 255. 10.1007/s42452-024-05932-z

[B50] Tao Z. Yuan H. Liu M. Liu Q. Zhang S. Liu H. (2023). Yeast extract: characteristics, production, applications and future perspectives. J. Microbiol. Biotechnol. 33 (2), 151–166. 10.4014/jmb.2207.07057 36474327 PMC9998214

[B51] Tripathi V. Gaur V. K. Dhiman N. Manickam N. (2020). Characterization and properties of the biosurfactant produced by PAH-Degrading bacteria isolated from contaminated oily sludge environment. Environ. Sci. Pollut. Res. 27, 27268–27278. 10.1007/s11356-019-05591-3 31190304

[B52] Wang J. Xu H. Guo S. (2007). Isolation and characteristics of a microbial consortium for effectively degrading phenanthrene. Pet. Sci. 4, 68–75. 10.1007/s12182-007-0012-y

[B53] Wulandari R. Lotrakul P. Punnapayak H. Amirta R. Kim S. W. Prasongsuk S. (2021). Toxicity evaluation and biodegradation of phenanthrene by laccase from *Trametes polyzona* PBURU 12. 3 Biotech. 11, 32. 10.1007/s13205-020-02556-z 33457166 PMC7782763

[B54] Zahed M. A. Matinvafa M. A. Azari A. Mohajeri L. (2022). Biosurfactant, a green and effective solution for bioremediation of petroleum hydrocarbons in the aquatic environment. Discov. Water. 2, 5. 10.1007/s43832-022-00013-x

[B55] Zargar A. N. Mishra S. Kumar M. Srivastava P. (2022). Isolation and chemical characterization of the biosurfactant produced by *gordonia* sp. IITR100. PloS ONE 17 (4), e0264202. 10.1371/journal.pone.0264202 35421133 PMC9009618

[B56] Zhao Z. Selvam A. Wong J. W. (2011). Synergistic effect of thermophilic temperature and biosurfactant produced by *Acinetobacter calcoaceticus* BU03 on the biodegradation of phenanthrene in bioslurry system. J. Hazard. Mater. 190 (1–3), 345–350. 10.1016/j.jhazmat.2011.03.042 21530078

[B57] Zhu X. Ni X. Waigi M. G. Liu J. Sun K. Gao Y. (2016). Biodegradation of mixed PAHs by PAH-Degrading endophytic bacteria. Int. J. Environ. Res. Public Health. 13 (8), 805. 10.3390/ijerph13080805 27517944 PMC4997491

